# Plant developmental stage influences responses of *Pinus strobiformis* seedlings to experimental warming

**DOI:** 10.1002/pei3.10055

**Published:** 2021-06-20

**Authors:** Ehren Reid Von Moler, Thomas Kolb, Anne Brady, Briana Nicole Palmiero, Taylor Robert Wallace, Kristen Marie Waring, Amy Vaughn Whipple

**Affiliations:** ^1^ Department of Biological Sciences Northern Arizona University Flagstaff AZ USA; ^2^ School of Forestry Northern Arizona University Flagstaff AZ USA; ^3^ Center for Adaptive Western Landscapes Northern Arizona University Flagstaff AZ USA

**Keywords:** acclimation, chlorophyll fluorescence, climate change, embryogenesis, growth tradeoffs, heat wave, in situ warming, oxidative stress, pine, seedling, water relations physiology

## Abstract

Seedling emergence, survival, morphological and physiological traits, and oxidative stress resistance of southwestern white pine (*Pinus strobiformis* Engelm.) were studied in response to warming treatments applied during embryogenesis, germination, and early seedling growth. Daytime air temperature surrounding cones in tree canopies was warmed by +2.1°C during embryo development. Resulting seeds and seedlings were assigned to three thermal regimes in growth chambers, with each regime separated by 4°C to encompass the wide range of temperatures observed over space and time across the species’ range, plus the effect of heat waves coupled with a high carbon emissions scenario of climate warming. The embryo warming treatment reduced percent seedling emergence in all germination and growth environments and reduced mortality of seedlings grown in the warmest environment. Warm thermal regimes during early seedling growth increased subsequent seedling resistance to oxidative stress and transpirational water use. Experimental warming during seed development, germination, and seedling growth affected seedling emergence and survival. Oxidative stress resistance, morphology, and water relations were affected only by warming imposed during germination and seedling growth. This work explores potential outcomes of climate warming on multiple dimensions of seedling performance and uniquely illustrates that plant responses to heat vary with plant developmental stage in addition to the magnitude of temperature change.

## 1 INTRODUCTION

A global mean temperature increase of 2.5℃ above pre‐industrial levels is possible by the year 2050 even under the lowest plausible relative concentration pathway (RCP 4.5; USGCRP, 2018). Regional climatic changes in the southwestern USA coupled with an increased prevalence of heat waves, however, are likely to exceed mean global temperature increases and exacerbate plant stress (Elias et al., 2018; Hultine et al., 2020; Mazdiyasni & Aghakouchak, 2015). This creates an urgent need to better understand the effects of climate warming on thermally sensitive forest regeneration processes including seed production, germination, and seedling establishment (Redmond et al., 2012; Seidel & Menzel, 2016; USGCRP, 2017, 2018). Long‐lived plant species are most vulnerable to temperature‐ and drought‐related mortality during the early seedling stage when rooting depth and volume, carbohydrate reserves, and the ability of seedlings to produce carbohydrates are limited (Hansen & Turner, 2019; Seidel & Menzel, 2016). Recent severe drought coupled with climate warming has contributed to forest tree dieback and reduced regeneration potential of conifers across the southwestern USA (Meddens et al., 2015; Minott & Kolb, 2020). Increasingly frequent and severe wildfires (Westerling et al., 2006) paired with reduced seedling establishment success increase the likelihood of transitions to novel ecosystem states (Davis et al., 2019). Undesirable ecosystem change may be promoted by high temperatures during seed maturation, germination, and early seedling growth, but an understanding of seedling responses to warming during early stages of plant development has remained elusive.

Acclimation to high temperature and related abiotic stresses during embryogenesis and early seedling growth may influence forest regeneration success with consequences for forest persistence during rapid climate warming (Seidel & Menzel, 2016). Plant acclimation to increased temperature is well documented (Al‐Hawija, Lachmuth, Welk, & Hensen, 2015; Kozlowski & Pallardy, 2002; Walter et al., 2013), but the relative impacts of warming during embryogenesis, germination, and seedling development on plant performance and survival under subsequent warming remains largely unknown. Acclimation refers to the process whereby plants are less negatively affected by a growth‐limiting environmental condition following repeated exposure to the limiting condition (Eyles et al., 2010). Stress‐induced cell signaling and modified gene expression and function may underlie altered plant performance following environmental stress exposure (Hossain et al., 2018; Lämke & Bäurle, 2017; Saini et al., 2019). Reactive oxygen species (ROS) and ROS‐regulating antioxidants constitute important components of plant oxidative stress systems that play a central role in cell signaling and plant responses to environmental stresses (Foyer & Noctor, 2005; Friedrich et al., 2019; Huang et al., 2019) including heat and drought (Abdallah et al., 2017; Wang et al., 2014). For instance, exposure of young winter wheat (*Triticum aestivum* L.) plants to temperatures of 32–34℃ produced an acclimation effect that increased antioxidant activity and was associated with improved plant growth under subsequent exposure to high temperatures (Wang et al., 2014). Additionally, epigenetic changes to the genome which can be heritable, have been shown to facilitate plant acclimation as well as adaptation by enhancing phenotypic exploration of fitness landscapes (see reviews in Richards et al., 2017; and Moler et al., 2018). For instance, epigenetic alterations of gene expression were found to underlie adaptive responses of phenological, growth, and cold hardiness traits in response to manipulated temperatures and photoperiods during embryogenesis of Norway spruce (*Picea abies*; Carneros et al., 2017; Johnsen et al., 2005; Yakovlev et al., 2011).

When parental environmental conditions are fine‐grained and unpredictable, greater phenotypic variation at the population level (i.e., parental “bet‐hedging”; Haaland et al., 2019) and optimized sensory and intercellular signaling mechanisms that enable adaptive responses to environmental stimuli may enhance progeny survival (Kuijper & Hoyle, 2015). The genome and the environment interact to determine phenotypic variation during plant development and growth, and phenotypic variation is constrained by resource availability as well as functional (e.g., trait tradeoffs) and genomic (e.g., pleiotropic) trait interdependence (Agrawal et al., 2010; Pigliucci, 2003; Reich et al., 2003; Simova‐Stoilova et al., 2016). Despite an increasing understanding of the mechanistic bases underlying acclimation, the extent to which acclimation to novel climates may aid species persistence during periods of rapid environmental change is poorly understood. While phenotypic plasticity may aid plant survival during rapid climate change (see review by Nicotra et al., 2010), it is unknown whether the potential for plants to acclimate to novel climates differs among the early, vulnerable stages of plant development.

We investigated southwestern white pine (*P*. *strobiformis*) seed sources from across Arizona and New Mexico to understand the effect of experimental heat exposure applied during embryogenesis, germination, and early seedling growth on seedling emergence, survival, and morphological and physiological traits. Southwestern white pine occurs from the southwestern USA throughout the Sierra Madre Occidental in Mexico (Shirk et al., 2018). The species is threatened by multiple disturbances including a lethal exotic fungal pathogen and increasing regional temperatures and drought (Breshears et al., 2005; Conklin et al., 2009; Geils et al., 2010; Shirk et al., 2018). We evaluated four hypotheses: (1) Morphological traits would be more strongly affected by warming applied during seedling growth whereas physiological traits would be responsive to warming applied during both seed maturation and seedling growth. This hypothesis follows from evidence that temperature‐induced shifts in biomass allocation are due to temperature sensing that occurs in roots and shoots that have, yet to develop before plants emerge from seeds (Bellstaedt et al., 2019; Feraru et al., 2019; Martins et al., 2017). Meanwhile, temperature changes during seed maturation and plant growth can influence cell chemistry with potential consequences for seedling physiology that may persist through the seedling stage (Carneros et al., 2017; Dafny‐Yelin et al., 2008). (2) Seedlings that survived warming during embryogenesis, germination, and seedling growth possess enhanced resistance to oxidative stress as well as increased transpiration, which can relieve heat stress‐induced limitations to photosynthesis via cooling (Radin et al., 1994). (3) Embryo warming would reduce the percentage of successful seedling emergence equally across temperature treatments because higher temperatures during seed development should stimulate a higher rate of basal metabolism and cause the developing embryo to more quickly consume seed‐borne reserves (Murray et al., 2004). (4) Emerged seedlings from embryo‐warmed seeds would exhibit decreased mortality, relative to unwarmed seeds, when grown in temperatures that exceed that of seed source sites. This follows from the assumption that embryo warming will have acted as a filter to exclude non‐heat‐tolerant phenotypes during germination and may have induced heat acclimation through higher rates of transpiration (Monteiro et al., 2016), or changes in cell chemistry that enhance cellular protection (Wang et al., 2014).

## 2 METHODS

### 2.1 Study overview

We investigated the responses of seedling emergence and survival, nine morphological traits, and eight physiological traits of *P*. *strobiformis* seedlings to a factorial combination of controlled warming treatments applied to plants before and after seed collection. Controlled warming treatments before seed collection consisted of passive warming treatments deployed in tree canopies. Controlled warming treatments after seed collection consisted of allowing plants to germinate and grow under three thermal regimes. Regimes were selected to represent plausible temperatures over the geographic range of *P*. *strobiformis* in the southwestern USA by the year 2050. We also conducted two additional experiments to understand how controlled warming influences seedling performance under oxidative stress induced by experimental drought and exposure to an herbicide that produces photooxidative damage (Ekmekci & Terzioglu, 2005).

### 2.2 Embryo warming treatments

**FIGURE 1:**
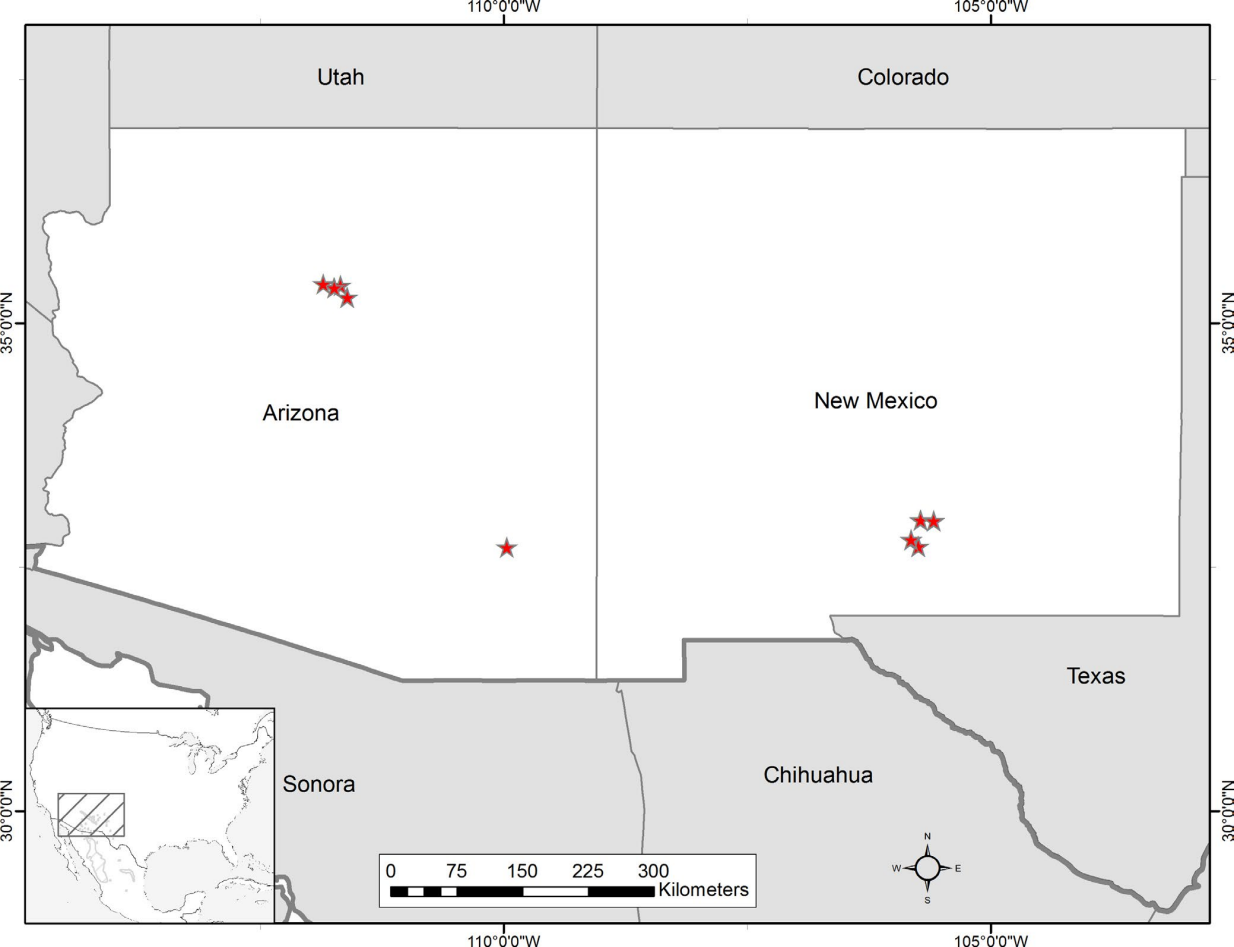
Map of sampled stands. Stand locations are marked by red stars

Embryo warming treatments with corresponding controls were deployed in 20 seed source trees from 10 stands across Arizona and New Mexico (*n* = 1–3 trees per stand; Figure 1). Following Ashton and Kelty (2018), we define stand as a contiguous group of trees occupying similar site conditions. The number of trees per stand was not held constant due to some stands containing a limited number of trees, low seed cone production, and inaccessible tree canopies. The within‐canopy embryo warming treatment was implemented by placing bags over developing seed cones to create a greenhouse effect, following Moler et al. (2021). The bags remained in‐place from late spring of the final year of seed and cone development until cone collection in late summer to early autumn (Table 1). The embryo warming treatment was timed to elevate air temperatures surrounding seed cones from the period of megagametophyte fertilization through the completion of seed maturation, encompassing a developmental period when warmer temperatures are known to alter later seedling phenology and gene expression (Carneros et al., 2017), and perhaps influence embryo development and maturation (Gruwez et al., 2014). Embryo warming deployments in each canopy consisted of three to five control bags and three to five treatment bags randomly distributed throughout all accessible aspects of the upper canopies of each tree. Each bag was affixed around at least two seed cones, as described in Moler et al. (2021). Embryo warming treatments were initiated in late April through early May during three years: 2014, 2015, and 2016. Cones were retrieved from September to October of the same year that treatments were deployed. Warming treatments achieved a +2.14℃ (±0.14 standard error of the mean) increase of daytime air temperatures surrounding developing seed cones. Briefly, the embryo warming treatment consisted of a combination of an insulative, nonporous bubble‐wrap packaging bag inside of a polyester pollination bag. The control treatment consisted of a high‐airflow porous mesh nylon bag. All bag types were deployed in tree canopies by loosely fitting bags around cones and affixing bags to tree branches with plastic cable ties placed over a ~5‐cm segment of polyethylene foam pipe insulation and secured onto branches with moderate pressure. Cones were bench‐dried in the greenhouse before seed extraction and stored at −10℃.

**TABLE 1:** Locations of seed source trees sampled for the present study. Embryo warming versus control treatments were established in all seed source trees, and seeds from all trees were randomly assigned to and grown in growth environments. Seed source trees included in additional treatments described in the text are demarcated with an “x”

National forest	Ranger district	Seed source tree	Latitude	Longitude	Tissue water relations	Paraquat treatment	Experimental drought *ψ* and *F′*
Coconino	Peaks	ATH1	35.38672	−111.67619	x		
Coconino	Peaks	ATH3	35.38670	−111.67476			
Coconino	Peaks	BIS3	35.36298	−111.73974	x	x	x
Coconino	Peaks	HP633	35.36410	−111.73692		x	
Coconino	Peaks	KEH503	35.40301	−111.85034		x	
Coconino	Peaks	UEL638	35.26414	−111.60625	x	x	
Coconino	Peaks	UEL893	35.26296	−111.60505	x		
Coronado	Safford	RIG291	32.70448	−109.96596	x		
Coronado	Safford	RIG295	32.70466	−109.96514	x		
Lincoln	Sacramento	BRA23	32.97790	−105.72024		x	x
Lincoln	Sacramento	BRA38	32.98066	−105.71640			
Lincoln	Sacramento	SAC260	32.71043	−105.74358			
Lincoln	Sacramento	SAC263	32.71125	−105.74149	x		
Lincoln	Sacramento	SAC264	32.71162	−105.74129	x		
Lincoln	Sacramento	SIX285	32.97166	−105.58348	x		
Lincoln	Sacramento	SIX287	32.97295	−105.58244			
Lincoln	Sacramento	SIX696	32.97292	−105.58259	x		
Lincoln	Sacramento	SUN204	32.77940	−105.81413	x		
Lincoln	Sacramento	SUN21	32.78060	−105.81413	x		
Lincoln	Sacramento	SUN647	32.78155	−105.81321	x		

### 2.3 Germination and growing conditions under experimental warming

Seeds were soaked in 1% H_2_O_2_ for 24 h, soaked in water for 24 h, and then cold stratified at 0.56℃ for 30 days before sowing in growth chambers. Seeds representing 20 genetic families from 10 stands (*n* = 2980) were sown into labeled RLC4 container growth tubes (Stuewe & Sons, Inc.; 2.5 cm diameter × 16 cm deep, 66 ml volume) filled with a loose granule clay soilless growth medium (Turface Pro League^®^ sports‐field conditioner) to facilitate separation of seedlings from growth media without loss of roots, and were topped with live soil inoculum gathered from *P*. *strobiformis* seed sources across Arizona and New Mexico. Seeds were randomly assigned to trays within growth chambers to ensure even replication of seedlings within warming treatments across seed source sites and genetic families (*n* = 14–33). Seedlings were sown on March 16, 2018 and immediately placed into one of three growth chamber thermal regimes. Destructive measures began on July 16, 2018, for a total of 122 days of exposure to assigned thermal regimes, including the periods of germination and early seedling growth. Growth chamber thermal regimes were set according to the assumption that seeds germinate at the onset of the summer monsoon season because we previously observed better germination at warm temperatures (unpublished data). Accordingly, the three growth chamber thermal regimes (hereafter referred to as growth environments) were 4℃ warmer during the first half than during the second half of the growth period. See Table 2 for thermal conditions of growth environments. Conceptually, the growth environments included (1) a cool temperature regime (closely matching temperatures at the most northerly sites from which seeds were collected), (2) a medium temperature regime (closely matching temperatures at more southerly sites from which seeds were collected), and (3) a warm temperature regime (exceeding temperatures at the warmest sites from which seeds were collected). The temperature of the warmest growth environment was 3.4℃ higher than the mean daily high temperature of the hottest month at the warmest seed collection site, and 7.4℃ higher than the mean daily high temperature of the hottest month at the coolest seed collection site. Thus, during the first 2 months of the experiment, seedlings in the warmest growth environment were exposed to temperatures exceeding the averages illustrated by climate norms. This large temperature level was chosen because carbon emissions scenarios since the year 2000 most closely resemble RCP 8.5, which may raise mean annual air temperatures in the southwestern USA by 4℃ by mid‐century (Elias et al., 2018), and by 6–9℃ by late‐century (Diffenbaugh & Field, 2013; USGCRP, 2018). Additionally, an increased duration and frequency of heat waves may lead to even further warming in the USA and abroad (Mazdiyasni & Aghakouchak, 2015; Mitchell et al., 2016). Growth environment temperatures were decreased 2 months following sowing to simulate the reduced temperature that follows monsoon season in southwestern North America. All growth environments received evenly distributed supplemental light (150 µmol × m^−2^ × s^−1^) at a 14‐h light/10‐h dark photoperiod for the first month following seed sowing. The photoperiod was decreased to 12‐h light/12‐h dark at the beginning of the second month following seed sowing to mimic shortening days. Seven weeks after seed sowing, fertilizer applications began at a rate of 15 ppm of Jack's Professional water soluble 20–20–20 general purpose fertilizer (J.R. Peters, Inc.) applied using a hand pump sprayer from approximately 5 cm above seedlings three times a week, on Mondays, Tuesdays, and Wednesdays. Fertilizer application rates were increased by 15 ppm weekly until a rate of 60 ppm was reached, at which point fertilizer was applied at a concentration of 60 ppm through the end of the growth period. We applied approximately 30 ml fertilizer solution to each seedling container per application. In addition to fertilizer applications, seedling foliage was misted with tap water via a hand pump sprayer once per day on Thursdays and Saturdays, and deep‐watered by evenly distributing 5.67 L of tap water across each container tray using a watering can on Fridays and Sundays to flush containers of accumulated salts. The watering and fertilization regime ensured that seedlings in all growth environments remained well watered despite the fast draining coarse media that was used. Media at the bottom of the growth containers was routinely spot checked for moisture by hand to confirm that water was consistently available to seedlings throughout the planting containers, as the presence of moisture on seedling container bottoms was possible only if water had moved vertically through the soil media. The small volume of the container (66 ml) relative to the volume of fertilizer solution applied (30 ml), coupled with the homogenous coarse‐grained planting media ensured that water was well distributed throughout the seedling containers. Additionally, when containers holding dead seedlings were emptied, the soil media was observed to be moist throughout the containers. To minimize microclimate effects, seedling container trays were rotated 180^◦^ and moved to the next higher rack within their respective growth environments weekly, at which time trays occupying top‐most racks were rotated to the bottom racks.

**TABLE 2:** Programmed thermal regimes of cool, medium, and warm growth environments

Month of growth	Cool (℃)		Medium (℃)		Warm (℃)	
	Night	Day	Night	Day	Night	Day
1–2	12	22	16	26	20	30
3–4	8	18	12	22	16	26

### 2.4 Emergence, morphology, and mortality

Seedling emergence and mortality were recorded daily. We also recorded: (1) the number of cotyledons produced, (2) the length of the longest cotyledon, (3) seedling height (as a measured from growth media to the topmost bud or base of the topmost needles), (4) diameter at root collar (DRC; measured with calipers as stem diameter directly above the growth media), and (5) seedling root and shoot dry mass (measured with an Ohaus Explorer^®^ balance following drying at 60℃ for 48 h). All destructive measures began on July 16, 2018 at the end of the 4‐month growing period, and ended on July 20, 2018, with the exception of a seedling subset included in an experimental drought (described below). The ratio of root‐to‐shoot mass was calculated for each plant. Seedling slenderness was calculated as shoot height divided by the DRC (Trubat et al., 2010).

### 2.5 Seedling water relations

#### 2.5.1 Tissue water relations

At 15 weeks of growth, 80 seedlings from 11 maternal trees representing six stands were tested for tissue water relations following Bongarten and Teskey (1986). Sample sizes ranged from *n* = 1–7 seedlings per stand × family × embryo warming × growth temperature treatment combination (equal to *n* = 5–30 seedlings per embryo warming × growth temperature treatment combination). The large range in sample sizes resulted from differences in the number of seedlings from each family included at the beginning of the study, and mortality of seedlings from different seed source and treatment combinations. Where possible, we included seedlings from all seed sources in measurements of water relations. We emphasize that a wide range of seed sources is represented in assessments of temperature treatments, and that we did not seek to investigate differences among seed sources within or among stands. Randomized seedling selections were instead designed to maximize the number of genetic families and evenness of replication across genetic families represented in water relations tests. Plants were taken from growth environments and severed at root collars. Shoots were submerged in tap water for 12 h to obtain seedling mass and stem water potential (*ѱ*_mid_) at full saturation. At 8 hourly intervals, *ѱ*_mid_ and seedling mass were measured using a portable pressure chamber (PMS Instrument Company^©^) and an Ohaus Explorer^®^ balance as seedlings were bench‐dried. Measurements ceased when *ѱ*_mid_ fell below −3.0 MPa, after which samples were dried at 65℃ for 48 h and weighed to obtain dry masses. From saturated sample mass, progressively drying sample mass and sample dry mass, relative water content (RWC) was calculated at the time of each *ѱ*_mid_ measurement as: fresh weight − dry weight/(saturated weight − dry weight) × 100 (Farrell et al., 2017; Lugojan & Ciulca, 2011). Turgor loss point and bulk modulus of elasticity values for each plant were calculated following common methods (Schulte & Hinckley, 1985; Turner, 1981). Specifically, the negative inverse of *ѱ*_mid_ was plotted against relative water content using the pressure‐volume curve calculator available at http://landflux.org/Tools.php, as described by Tu (2008) and Farrell et al. (2017).

#### 2.5.2 Whole plant hydraulic resistance (*R*_root + stem_)

At 15 weeks after seed sowing, 195 seedlings representing 14 maternal trees from seven stands were tested for differences in *R*_root + stem_ across the embryo warming and seedling growth temperature treatments. Sample sizes ranged from *n* = 1–7 seedlings per stand × family × embryo‐warming × growth temperature treatment combination (equal to *n* = 16–66 seedlings per embryo‐warming × growth temperature treatment combination). Once again, randomized seedling selections were designed to maximize the number of seed sources, not to allow testing for differences among seed sources. Methods of calculating *R*_root + stem_ were based on Baert et al. (2015), using the following equation:

$$R_{\text{root}+\text{stem}}=\frac{\psi_{s}‐\psi_{w}}{E}$$

where *ψ*_s_ = water potential of saturated soil, *ψ*_W_ = stem water potential (as above), and *E* = foliar transpiration measured at mid‐day using an infrared gas exchange analyzer (Optisciences^©^). Following *E* measurements, seedlings were cut at the root collar to determine seedling *ψ*_mid_. Water potential of saturated soilless growth media was assumed to be 0.001 MPa to account for the low matric potential of the coarse soilless growth media and approximate the theoretical water potential of a saturated soil in which soil water is 100% available to plants (O’Geen, 2013).

### 2.6 Induced oxidative stress and chlorophyll *a* fluorometry

Oxidative stress due to paraquat exposure and natural causes can be measured as a reduction of the light‐adapted quantum efficiency of open reaction centers of PSII (i.e., *F′*, measured via chlorophyll *a* fluorometry; Ekmekci & Terzioglu, 2005), calculated as follows:

$$F^{\prime }=\frac{F_{m}^{\prime }‐F_{s}}{F_{m}^{\prime }}$$

where *F*′_m_ is the maximum fluorescence of chlorophyll *a* during a saturated light pulse and *F*_s_ is stable state fluorescence of chlorophyll *a*. *F′* indicates the closure of PSII reaction centers due to excessive excitation energy and heat dissipation caused by nonphotochemical quenching, and is a reliable assessment of oxidative damage to PSII (Maxwell & Johnson, 2000) due to heat (Haldimann & Feller, 2004) and drought (Cavender‐Bares & Bazzaz, 2004). Fuerst and Vaughn (1990) reported that the resistance of ten weed species to paraquat was due either to enhanced oxidative stress resistance, or to rapid sequestration of paraquat from the chloroplast, which in *Conyza* sp. resulted in a failure of paraquat to penetrate chloroplast membranes (Fuerst et al., 1985). Therefore, change in *F*′ or the development of lesions upon paraquat exposure does not offer a foolproof stand‐alone measure of oxidative stress for all plant species, though it has indeed been found to provide accurate approximations of plant oxidative stress in the studies cited above, and the effective quantum yield of photosynthesis (*F′*) has been shown to reliably detect oxidative damage sustained by PSII due to paraquat (Maxwell & Johnson, 2000).

We measured *F′* from a random sample of seedlings (*n* = 153) from 7 stands and 14 seed source trees during the last week of the 4‐month growth period to determine reference *F′* values with respect to embryo warming and growth environment. To assess seedling resistance to artificial oxidative stress via paraquat exposure, 258 seedlings representing five seed source trees, each from a separate stand, were moved to a fume hood and randomly assigned to either a paraquat treatment group (*n* = 136), or RO water control group (*n* = 122) with treatment sample sizes of *n* = 1–16 seedlings per stand × family × embryo‐warming × growth environment combination; *n* = 28–53 seedlings per embryo‐warming × growth environment combination (Table 3). Paraquat induces oxidative stress in plants only after plants are exposed to direct sunlight following foliar absorption of paraquat, and thus seedlings were either subjected to paraquat +sunlight during mid‐day, or RO water +sunlight during mid‐day according to the group to which they were randomly assigned. The paraquat treatment and RO water control treatment consisted of three successive exposures of seedlings to either a 5 mM of paraquat solution or RO water. Exposure to paraquat or water occurred over the course of 2 days beginning during the afternoon of day 1 and concluded during the morning of day 2. Paraquat exposure #1: seedling foliage was submerged in 5 mM paraquat for 30 s during the afternoon of the first day. Paraquat exposure #2: a cotton swab soaked in 5 mM paraquat, flattened by stretching to increase surface area, was draped over seedling foliage during late afternoon of the first day and remained on seedlings overnight to allow further time for foliage to absorb paraquat. Paraquat exposure #3: seedling foliage was submerged in 5 mM paraquat for 10 s during the morning of the second and final day of the paraquat and RO exposure treatment. Plants assigned to the control group were treated as above but with RO water instead of paraquat solution. Following the final paraquat exposure, seedlings were exposed to direct sunlight outdoors at an altitude of approximately 2133 m for 1 h at approximately 09:00 h followed by 30 min of direct sunlight exposure outside at approximately 14:00 h. Seedlings were then left under a full‐spectrum LED lamp (~150 µmol/m^2^/s) for 16 h before a final exposure to direct sunlight outside for 1 h at approximately 09:00 h. Light‐adapted chlorophyll fluorescence was measured using an Optisciences^©^ iFL unit immediately after sunlight exposure at 09:00 h on both days. Paraquat‐treated and control seedlings were measured in alternating succession to ensure that time elapsed since light exposure did not bias results.

**TABLE 3:** Model summaries of predictors of response variables, emphasizing those not provided in text. SE is standard error of the mean. Estimate is the estimated *F*‐test coefficient. *df* is denominator‐degrees of freedom. **Control** refers to the non‐embryo‐warmed treatment level. TLP is turgor loss point. **High** growth environmental temperature level and **embryo warming treatment** are reference factor levels in models

Response	Predictor	Estimate	SE	*df*	Test statistic	*p*‐value
Proportion mortality	Intercept	−1.40	0.40	1475	−3.49 (*z*)	4.9E‐4
Proportion mortality	Cool environment	−1.66	0.212	1475	7.86 (*z*)	3.9E‐15
Proportion mortality	Medium environment	−1.48	0.199	1475	7.44 (*z*)	1.0E‐13
Proportion mortality	Family	NA	NA	NA	0.36 (LRT)	0.55
Days to mortality	Intercept	3.56	0.12	182	29.06 (*z*)	<2.0E‐16
Days to mortality	Cool environment × Control × Seed mass	−4.89	1.14	182	−4.301 (*z*)	1.7E‐5
Days to mortality	Medium environment × Control × Seed mass	−3.29	90	182	−3.64 (*z*)	2.7E‐4
Days to mortality	Medium environment × Seed mass × Climate	−0.23	0.06	182	−3.84 (*z*)	1.3E‐4
Days to mortality	Control × Seed mass × Climate	−0.16	0.034	182	−4.79 (*z*)	1.7E‐6
Days to mortality	Family	NA	NA	NA	147.99 (LRT)	<2.2E‐16
Proportion emerged	Intercept	−6.17	0.36	2949	−17.10 (*z*)	<2.0E‐16
Proportion emerged	Low environment	0.42	0.13	2949	3.30 (*z*)	9.5E‐4
Proportion emerged	Medium environment	0.26	0.12	2949	2.062 (*z*)	0.04
Proportion emerged	Control	−0.27	0.11	2949	−2.55 (*z*)	0.01
Proportion emerged	Climate PC1	0.04	0.02	2949	1.93 (*z*)	0.05
Proportion emerged	Family	NA	NA	NA	263.61 (LRT)	<2.2E‐16
24 h PQ *F′*	Intercept	0.76	.02	244	36.32 (*t*)	<2.0E‐16
24 h PQ *F′*	Paraquat control × Cool environment	−0.11	0.033	244	−3.24 (*t*)	1.4E‐3
24 h PQ *F′*	Paraquat control × Medium environment	−0.05	0.030	244	−1.50 (*t*)	0.14
24 h PQ *F′*	Family	NA	NA	NA	2.84 (LRT)	1
48 h PQ *F′*	Intercept	0.76	0.03	224	12.11 (*t*)	<2.2E‐16
48 h PQ *F′*	Paraquat control × Cool environment	−0.19	0.038	224	−4.90 (*t*)	1.8E‐6
48 h PQ *F′*	Control × Seed mass	0.79	0.25	224	3.21 (*t*)	1.5E‐2
48 h PQ *F′*	Family	NA	NA	NA	2.25 (LRT)	1
Cotyledon length	Intercept	1.28	0.04	1248	36.17 (*t*)	<2.0E‐16
Cotyledon length	Cool environment	−0.03	0.01	1248	−2.43 (*t*)	0.02
Cotyledon length	Medium environment	0.06	0.01	1248	4.81 (*t*)	1.7E‐6
Cotyledon length	Family	NA	NA	NA	18.23 (LRT)	<0.01
Day 10 *ψ* water	Intercept	38.94	7.54	33	5.17 (*t*)	<2.0E‐16
Day 10 *ψ* water	Cool environment	−18.82	3.44	33	−5.47 (*t*)	4.7E‐6
Day 10 *ψ* water	Medium environment	−15.65	3.53	33	−4.43 (*t*)	9.7E‐5
Day 10 *ψ* water	Family	NA	NA	NA	<0.01 (LRT)	0.99
DRC	Intercept	1.05	0.03	1268	35.07 (*t*)	<2.0E‐16
DRC	Cool environment	0.24	0.01	1268	17.56 (*t*)	<2.0E‐16
DRC	Medium environment	−0.01	0.01	1268	−0.49 (*t*)	0.62
DRC	Family	NA	NA	NA	7.69 (LRT)	<0.01
*E*	Intercept	2.58	0.17	178	15.03 (*t*)	<2.0E‐16
*E*	Cool environment	−0.85	0.23	178	−3.76 (*t*)	<0.01
*E*	Medium environment	−0.42	0.18	178	−2.38 (*t*)	1.8E‐2
*E*	Family	NA	NA	NA	1.26 (LRT)	0.99
Root:Shoot mass	Intercept	−0.74	0.05	894	−16.12 (*t*)	<2.0E‐16
Root:Shoot mass	Cool environment	0.33	0.04	894	7.52 (*t*)	1.32E‐13
Root:Shoot mass	Medium environment	0.01	0.04	894	0.33 (*t*)	0.74
Root:Shoot mass	Family	NA	NA	NA	4.3E‐07 (LRT)	0.99
Root mass	Intercept	−3.54	0.10	1144	−35.13 (*t*)	2.2E‐16
Root mass	Cool environment	0.61	0.05	1144	11.93 (*t*)	2.2E‐16
Root mass	Medium environment	0.12	0.05	1144	2.30 (*t*)	2.16E‐2
Root mass	Family	NA	NA	NA	6.65 (LRT)	9.9E‐3
RWC	Intercept	0.49	0.08	66	6.17 (*t*)	4.7E‐8
RWC	Cool environment	0.06	0.06	66	1.11 (*t*)	0.27
RWC	Medium environment	0.16	0.05	66	3.43 (*t*)	1.06E‐3
RWC	Family	NA	NA	NA	32.14 (LRT)	<0.01
Shoot mass	Intercept	0.04	0.01	904	4.46 (*t*)	9.2E‐06
Shoot mass	Cool environment	0.03	0.005	904	7.48 (*t*)	1.8E‐13
Shoot mass	Medium environment	0.01	0.005	904	2.77 (*t*)	0.01
Shoot mass	Family	NA	NA	NA	9.80 (LRT)	1.7E‐3
Slenderness	Intercept	4.55	0.12	1269	39.43 (*t*)	<2.2E‐16
Slenderness	Cool environment	−0.49	0.077	1269	−6.34 (*t*)	3.27E‐10
Slenderness	Medium environment	0.39	0.077	1269	5.01 (*t*)	6.08E‐7
Slenderness	Family	NA	NA	NA	74.03 (LRT)	<0.01
Leaf conductance	Intercept	0.10	0.001	154	17.01 (*t*)	<2.2E‐16
Leaf conductance	Cool environment	−0.03	0.008	154	−4.30 (*t*)	3.08E‐5
Leaf conductance	Medium environment	−0.03	0.006	154	−4.93 (*t*)	2.08E‐6
Leaf conductance	Family	NA	NA	NA	1.58E‐9 (LRT)	1
Total dry mass	Intercept	−2.42	0.08	893	−30.24 (*t*)	<2.2E‐16
Total dry mass	Cool environment	0.38	0.040	893	9.58 (*t*)	< 2.2E‐16
Total dry mass	Medium environment	0.11	0.040	893	2.61 (*t*)	0.01
Total dry mass	Family	NA	NA	NA	9.90 (LRT)	1.7E‐3
*Non‐significant physiology variables*						
**Response**	**Fixed effect predictor**	***χ* ^2^**	***df***	***p*‐value**		
*ψ*_O_	Growth environment	1.62	2	0.44		
*ψ*_O_	Embryo warming	1.81	1	0.18		
BME	Growth environment	4.33	2	0.14		
BME	Embryo warming	2.22	1	0.99		
*ψ*_TLP_	Growth environment	0.00	2	0.99		
*ψ*_TLP_	Embryo warming	0.09	1	0.77		
*R*_root + stem_	Growth environment	4.21	2	0.12		
*R*_root + stem_	Embryo warming	0.76	1	0.38		

### 2.7 Light‐adapted chlorophyll fluorescence (*F′*) responses to experimental drought and induced oxidative stress

Chlorophyll fluorescence is a useful indicator of damage to photosystem II resulting from biotic (Pérez‐Bueno et al., 2019) and abiotic stress (Baker, 2008). To determine whether *P*. *strobiformis* seedling *F′* responses to paraquat exposure can be used to assess plant resistance to ecologically realistic oxidative damage, we compared *F′* values of droughted seedlings to those of seedlings exposed to paraquat. We measured *F′* values for the following three experimental groups: (1) plants exposed to paraquat and light to achieve oxidative stress (*n* = 135), (2) plants not exposed to paraquat or experimental drought (control group; *n* = 122), and (3) non‐paraquat‐treated plants exposed to 10 days of experimental drought (*n* = 39). At 10 days of experimental drought, 18% of the droughted seedlings exhibited mid‐day stem water potential (*ѱ*_mid_) values ≤−3.0 MPa, and the average *ѱ*_mid_ was −1.4 MPa.

### 2.8 Statistical analyses

All analyses were conducted in the R statistical program (version 3.6.2, R Core Team, 2018). Related R code and datasets are available through the online Knowledge Network for Biocomplexity (KNB). Linear mixed effect models in the packages *nlme* and *glmmTMB* were used for all statistical tests of significant differences among mean trait values and tests of interactions among fixed factors and covariates. Fixed effects in the model were: growth environment, embryo warming treatment, seed mass, and the first principal component of climate variables from seed source environments. The first principal component (PC1) was included as a model covariate to maximize the amount of model error explained by seed source climates using only one dimension of climate data. Climate data were accessed through ClimateWNA (Wang et al., 2016). Principal component axes were derived from 30 years norms of 235 variables describing annual, seasonal, and monthly climate spanning the period 1980–2010. PC1 explained 74.2% of variation in the full climate dataset (Figure S1). The 65 largest eigenvalues of PC1 all describe thermal properties of seed source sites (Table S1). Degree days above 18℃ in March was the largest eigenvalue of PC1 (Table S1). The maximum temperature in September was the largest eigenvalue of PC2 and mean annual precipitation was the fifth largest eigenvalue of PC2 (Table S1), which explained 11.3% of variation in climate data. Precipitation during September was the top contributor to PC3 (Table S1), which explained 7.0% of variation in climate data. Temperature is more likely than precipitation to vary across the relatively small spatial extents within the three regions of the southwestern USA where samples were collected. The dominance of PCs by temperature‐related variables rather than variables related to precipitation likely follows from the clustered distribution of seed source sites within the three regions in which stands were sampled (Figure 1). In all mixed‐effect models, seed source tree nested within stand was included as a random effect. The number of days between seed sowing and mortality were used for tests of the timing of mortality. Binomial distributions were specified in mixed‐effect models of seedling mortality and emergence. Poisson distributions were specified in mixed‐effect models of the number of days‐to‐seedling mortality. Data transformations included log_10_ or square root transformations depending on the variable (details available in code deposited in KNB), and were applied to continuous response variables in a limited number of cases to satisfy the assumption of normal distribution of residuals.

For all models, model structure and covariates including seed source climate and seed mass were assessed for parsimony by comparing Akaike information criterion (AIC) scores of contrasting models. If significant interactions were detected, interactive models were included in model comparisons using AIC. Models with the smallest AIC were favored, and when competing models were within 2 AIC units of the smallest AIC, the simplest model structure with the least covariates was selected for hypothesis testing. For example, in the case of an additive model with an AIC score 1.99 larger than a model with interaction terms, the simpler additive model would be selected for subsequent hypothesis testing. AIC scores for each model compared are provided in Table S2. Satterthwaite approximation of denominator degrees of freedom was specified for all omnibus F‐tests of fixed effects as well as type III sum of square ANOVAs for models that included interactions, whereas Type II sum of square ANOVAs were specified for additive models. A significant interaction between embryo warming, seed mass, and seed source climate precluded post hoc testing for contrasts within factor levels of growth environment and seed warming. Significance of random effects was evaluated using likelihood ratio (chi‐squared distribution) testing.

## 3 RESULTS

### 3.1 Seedling morphological and physiological responses to warming during embryogenesis and growth

**FIGURE 2:**
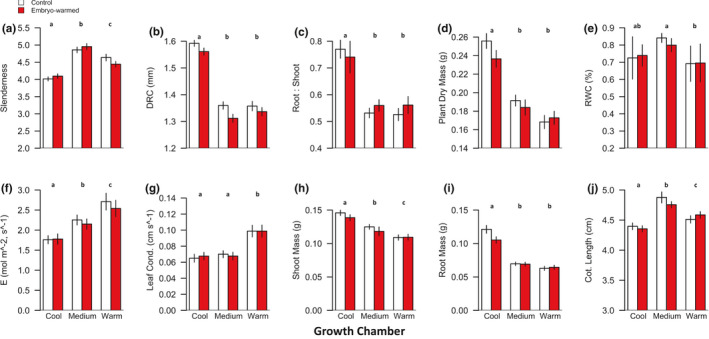
(a–j) Red bars are mean measures of seedlings from the seed warming treatment. White bars are mean seedling measures of seedlings from the control treatment. Lower case letters over bars indicate significant differences among growth environments (Tukey HSD post‐hoc contrasts, *p* < 0.05). ±1 SE shown. DRC = diameter at root collar. RWC = relative water content. E = foliar transpiration. Leaf cond. = leaf (stomatal) conductance. Cot. Length = mean cotyledon length

Seven morphological traits (DRC, root mass, shoot mass, root:shoot mass, total dry mass, slenderness, and cotyledon length) and three physiological traits that describe tissue water relations, and gas exchange (RWC, leaf conductance, and *E*) varied significantly among growth environment temperature treatments (*p* < 0.05, Figure 2a–j; Table 3). We observed a general trend of reduced seedling size and mass in the medium and warm growth environments coinciding with higher *E* and leaf conductance at warmer temperatures (Figure 2). Neither water potential at turgor loss point, bulk modulus of elasticity, nor osmotic potential at full turgor varied with embryo warming or growth environment temperature (Table 3). All morphological traits except root:shoot mass varied significantly across seed source trees (families), but RWC was the only tissue water relations trait that varied significantly across families (*p* < 0.05, Table 3).

### 3.2 Seedling *ѱ*_mid_ and *F*′ under drought and artificial oxidative stress

**FIGURE 3:**
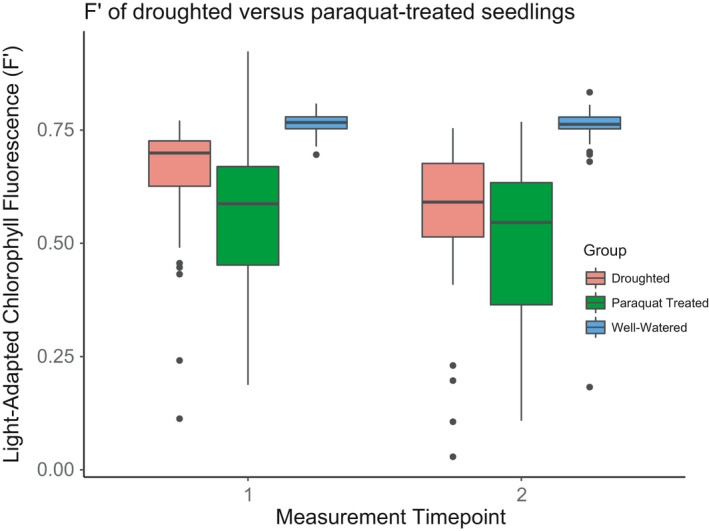
Comparison of two types of abiotic stress on light‐adapted chlorophyll fluorescence (*F′*) values measured at two timepoints. As noted in the main text, *F′* values above 0.7 indicate a functioning PSII, whereas values less than 0.7 indicate stress damage to PSII. Paraquat‐ (green filled boxes) or water‐treated (control; blue filled boxes) seedlings 24 h (Measurement Timepoint 1) and 48 h (Measurement Timepoint 2) after solution and sunlight exposure. Seedlings subjected to water withholding eight days (Measurement Timepoint 1) and ten days (Measurement Timepoint 2) after water withholding commenced (pink filled boxes)

**FIGURE 4:**
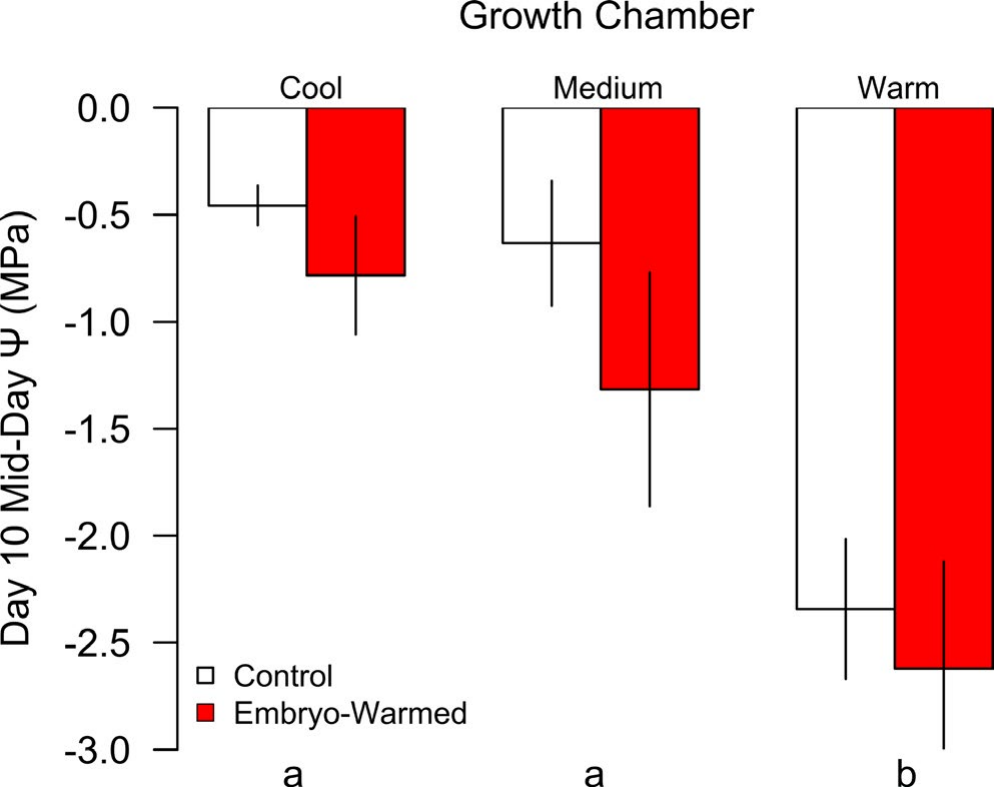
Mid‐day stem water potential (*ψ*) of seedlings subjected to embryo warming versus control treatments, grown for 4 months under three thermal regimes, then exposed to 10 days of experimental drought in the medium‐temperature environment. Red bars are mean measures of seedlings from the seed warming treatment. White bars are mean measures of seedlings from the control, that is, non‐seed‐warmed, treatment. Lower case letters below bars indicate significant differences among growth environments (Tukey HSD post‐hoc contrasts, *p* < 0.05). ±1 SE shown

**FIGURE 5:**
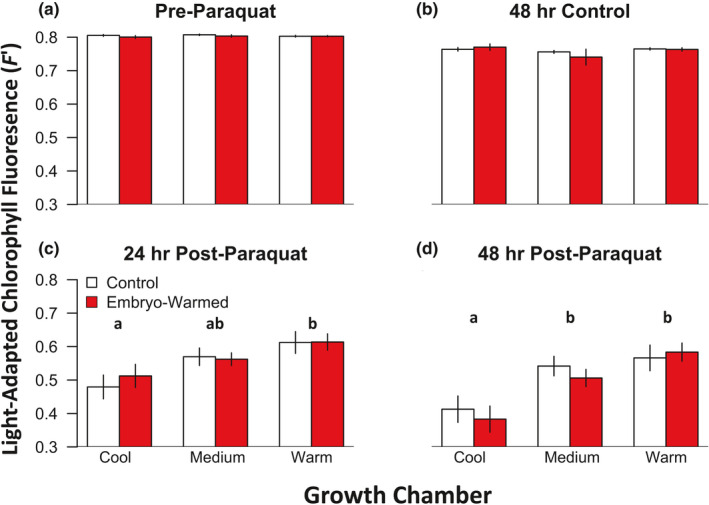
Light‐adapted chlorophyll fluorescence (*F′*) above 0.7 indicate a functioning PSII, and abiotic stress from natural and artificial sources can damage PSII thus causing reductions in *F′*. Red bars are mean measures of seedlings from the seed warming treatment. White bars are mean measures of seedlings from the control treatment. Lower case letters over bars indicate significant differences among growth environments (Tukey HSD post‐hoc contrasts, *p* < 0.05). No differences in *F′* were found between embryo warming treatment versus control or growth environments for seedling *F′* values measured before paraquat exposure (panel a, Pre‐Paraquat) or 48 h after exposure to RO water (panel b, 48 h Control). However, differences in *F'* among embryo warming treatments increased with time after paraquat exposure *(panels c and d)*. ±1 SE shown.

Seedlings subjected to oxidative stress via paraquat and via experimental drought had lower *F′* values than controls, but paraquat produced lower *F′* values than experimental drought (Figure 3). Seedling *F′* and *ѱ*_mid_ both recorded on the 10th day of experimental drought were significantly correlated (*r* = 0.35, *p* = 0.016). Values of *ѱ*_mid_ on the 10th day of experimental drought varied significantly with growth temperature, with a positive relationship between growth environment temperature and *ѱ*_mid_ (*χ* = 34.3, *p* < 0.001, Table 3; Figure 4). Seedling *F′* measures conducted before paraquat exposure and 48 h after RO water‐treatment (control) did not vary with embryo warming or growth temperatures (Figure 5a,b). *F′* values from non‐paraquat‐treated seedlings were >0.75, within the range of *F′* values expected for healthy plants (Guidi et al., 2019), and thus can be used as a reference *F′* indicative of an undamaged PSII for *P*. *strobiformis* seedlings. Mean seedling *F′* values measured 24 h after exposure to paraquat and full sunlight were below 0.7 for all experimental warming groups, and 24 h *F′* values varied significantly with an interaction between growth environment and paraquat treatment (*χ* = 10.5, *p* = 0.005, Table 3; Figure 5c). Seedling *F′* values measured 48 h after exposure to paraquat and sunlight were lower than those measured after 24 h for all experimental warming groups, and 48 h *F′* values also varied significantly with an interaction between growth environment and paraquat treatment (*χ* = 26.0, *p* < 0.001). Seedling *F′* values measured 48 h after exposure to paraquat and sunlight also varied significantly in omnibus tests of significance with an interaction between seed mass, embryo warming, and paraquat treatment (*χ* = 10.3, *p* = 0.001, Table 3; Figure 5d).

### 3.3 Emergence and survival responses to warming treatments

**FIGURE 6:**
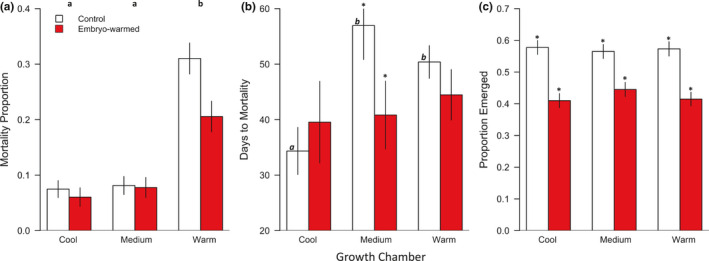
(a–c) Seedling emergence and survival as a function of embryo warming and temperature during seedling growth. Red bars are mean measures of seedlings from the embryo‐warming treatment. White bars are mean measures of seedlings from the control treatment. Lower case letters over bars indicate significant differences among growth environments; lower case italicized letters indicate significant differences among only means of seedlings from un‐warmed but not warmed embryos; asterisks indicate significant differences between seedlings from un‐warmed and warmed embryos within a growth environments (Tukey HSD post‐hoc contrasts, *p* < 0.05). ±1 SE shown

Percent mortality varied significantly and positively with temperature of growth environments (*χ* = 91.7, *p* < 0.001, Figure 6a). Specifically, the likelihood of seedling mortality in the warm environment was 81% higher as compared to seedlings grown in the cool environment, and 77% higher for seedlings grown in the medium temperature environment as compared to seedlings grown in the cool environment. Marginal statistical support was found for a 38% decreased likelihood of mortality when embryos were warmed (*χ* = 3.6, *p* = 0.058), and there was no support for the influence of interactions between growth environment temperature and embryo warming on seedling mortality. Days‐to‐mortality varied significantly with interactions both between growth environment, seed mass, and climate (*χ* = 15.3, *p* < 0.001, Figure 6b; Table 3), and between embryo warming, seed mass, and climate (*χ* = 23.0, *p* < 0.001, Table 3). Seed source family did not contribute to variation in percent mortality, but was a significant source of variation for days‐to‐mortality (*χ* = 148.0, *p* < 0.001). The likelihood of seedling emergence was 93% higher for control versus embryo‐warmed seedlings (*z* = 8.4, *p* < 0.001), and did not vary across growth environments (Figure 6c). We observed a modest but significant trend toward slower emergence in the warmest growth environment, wherein the mean difference in emergence timing among growth environments totaled to approximately 1 day (*χ* = 9.3, *p* = 0.01, range = 1–28 days until emergence). Seed source tree was a significant source of variation for both percent seedling emergence (*χ* = 253.6, *p* < 0.001) and days to emergence (*χ* = 14.8, *p* < 0.001).

## 4 DISCUSSION

Hypothesis (1) Morphological traits would be most responsive to warming during seedling growth, whereas physiological traits would not be responsive to warming during any one developmental stage.

Results did not support Hypothesis (1). Embryo warming resulted in notable effects for percent seedling emergence, percent mortality, and days to mortality. Morphological and physiological traits, on the other hand, exhibited sensitivity to the temperature of seedling growth environments. We observed declines in shoot mass, root mass, DRC, and the ratio of root‐to‐shoot mass in warmer growth environments. Abundant watering during seedling growth may have prompted plants to prioritize aboveground growth at the expense of belowground growth. And given that water, nutrient, and light levels were held constant across growth environments, and *F′* did not vary across growth environments, reduced total plant biomass in warmer growth environments was likely simply a result of an increased proportion of energy diverted from growth to basal metabolism in warmer temperatures (Clarke & Fraser, 2004). Alternatively, reduced biomass at higher temperatures may also have resulted from increased investment in cellular defense chemistry, such as antioxidants, at the expense of biomass accumulation (e.g., a growth‐defense tradeoff; Huot et al., 2014). Differential investment in cellular defense chemistry across temperature treatments may account for why differences in *F′* values among temperature treatments were not observed until plants were subjected to either paraquat or severe drought. Previous studies reported that plants grown under warmer temperatures exhibited increased antioxidant concentrations and activities against ROS (Wang et al., 2003, 2014). Similarly, we observed that seedlings grown in warmer environments expressed greater oxidative stress resistance than seedlings grown in cooler environments. However, since we did not measure antioxidant concentrations, we cannot confirm nor reject a role for differential investment in cellular chemistry across growth environments.

Hypothesis (2) Seedlings that survived warming during embryogenesis, germination, and seedling growth either possessed or were induced to express enhanced resistance to oxidative stress as well as increased transpirational cooling.

Results supported Hypothesis (2). A consistent effect of seed warming on *F′* values was not observed following exposure to paraquat, but oxidative stress (i.e., declines in *F′*) occurred less rapidly for seedlings subjected to warmer growth environments. Because paraquat damages PSII through oxidative stress, our results allow us to deduce that controlled warming during seedling growth improved seedling oxidative stress resistance. Warming has been found to influence photosynthetic thermotolerance in a manner that may improve protection of photosynthetic machinery against subsequent abiotic stress exposure (Ahuja et al., 2010; Leverenz et al., 1999; Nievola et al., 2017; Wang et al., 2003). And as noted above, we speculate that changes in antioxidant concentration resulting from warming in the present study may have resulted in the *F′* differences we observed among seedlings from different growth environments. Wheat plants (*Triticum aestivum*) exhibited increased thermotolerance and increased protection of PSII, which coincided with increased concentrations of antioxidants following heat acclimation treatments at 40℃ (Sharma et al., 2015). Additionally, wheat plants that maintained high levels of the quantum efficiency of PSII under heat stress also maintained high leaf conductance and *E* (Sharma et al., 2015), as we found in our study. A drought acclimation study of olive (*Olea europaea* L.; cultivar ‘Chétoui’) found that drought acclimation improved plant performance under subsequent drought events, and that drought‐induced increases in antioxidant concentrations declined soon after drought exposure (Abdallah et al., 2017). In olive plants, increased antioxidant concentrations may be a proximal but not an ultimate source of the drought acclimation trend reported (Abdallah et al., 2017), which provides support for the known role of antioxidants in cellular signaling. In the present study, variation in *F′* values related to temperature treatments was not apparent until imposition of artificial oxidative stress, which suggests that warming of *P*. *strobiformis* triggered an inducible mechanism of photoprotection.

Unexpectedly, we found evidence that warming during seedling development causes changes in seedling water relations that may predispose seedlings to drought stress even after temperatures are reduced. Seedlings reared in the warmest growth environment prior to experimental drought had significantly more negative *ѱ*_mid_ than seedlings reared in the cool‐ or medium‐temperature growth environments even after seedlings occupied a common thermal environment for 10 days during the drought treatment. The more negative *ѱ*_mid_ of seedlings grown in the warmest growth environment suggests a greater imbalance between *E* and water supplied to leaves than for seedlings grown in the cooler environments. This may be due to greater leaf conductance of seedlings from the warmest growth environment. While we expected to observe a positive relationship between growth environment temperature and leaf conductance (Urban et al., 2017), it is notable that plants grown in the warm environment continued to transpire more water even after warm‐grown plants were moved to the medium‐temperature growth environment for the experimental drought treatment. If this trend were to occur also in natural environments, undue seedling mortality could result from brief periods of anomalously high temperature and ample water during seedling development that precede dry periods. Embryo warming, on the other hand, did not influence seedling water use under experimental drought. We note that seedling development in the warmest growth environment, which predisposed seedlings to more negative *ѱ*_mid_, also coincided with increased oxidative stress resistance. Possible mechanisms driving the apparent relationship between increased water use and increased oxidative stress resistance; however, remain unclear.

Hypothesis (3) Embryo warming would reduce the percentage of successful seedling emergence equally across germination temperature treatments.

Results supported Hypothesis (3). This suggests that higher temperatures during seed development stimulate a higher rate of basal metabolism, which in turn may cause the developing embryo to more quickly consume seed‐borne reserves (Murray et al., 2004). In a study of *Glycine* taxa from across Australia, Murray et al. (2004) found that seed mass was greatest at the warmest seed source sites. Their study led them to conclude that larger seeds had more metabolizable reserves than smaller seeds, and that extra metabolizable reserves were important in warmer areas where basal metabolism occurs at a higher rate, without which seeds might not have sufficient reserves for germination and seedling establishment.

Hypothesis (4) Emerged seedlings from embryo‐warmed seeds would exhibit decreased mortality relative to unwarmed seeds when grown in environments warmer than seed source sites.

Results supported Hypothesis (4). Mortality did not increase incrementally with growth environment temperature, and instead rose precipitously between the medium and warm growth environments. Based on these findings, it appears that any potential benefit that may follow from seed maturation under the warmer temperatures of the embryo warming treatment (+2.1℃) may not be realized unless temperatures during seedling growth greatly exceed average source site temperatures, which may occur more frequently with climate change. We suspect that warming during seed maturation may have rendered inviable all seeds that were not genetically predisposed to heat tolerance, and additionally may have facilitated acclimation of seeds to warmer growth conditions through epigenetic mechanisms such as those found in *Picea abies* described earlier (Johnsen et al., 2005).

This study established that multiple commonly measured metrics of plant water relations (osmotic water potential (*ψ*_O_), bulk modulus of elasticity (BME), hydraulic resistance (*R*_root + stem_), water potential at the turgor loss point (*ψ*_TLP_)) were not affected by experimental warming during embryogenesis or seedling growth. The exceptions included the leaf‐level gas exchange responses of *E* and leaf conductance, and mid‐day water potential. However, our findings suggest that consequences of warming treatments for tissue water relations might become apparent once plants are exposed to drought, as we found in our assessment of *F′*, which did not vary across warming treatments until seedlings were subjected to oxidative stress.

Our finding that *P*. *strobiformis* seedlings achieve smaller sizes above‐ and belowground and a smaller root:shoot mass ratio when warmed during germination through early seedling growth suggests that *P*. *strobiformis* may be more likely to be shaded by competing vegetation and may have restricted access to soil water when growing in warmer conditions in natural settings. We note, however, that effects of warming independent of drought, as we describe here, may not be indicative of the responses of *P*. *strobiformis* seedlings simultaneously exposed to warming and drought. For example, a previous greenhouse study of *P*. *strobiformis* seedlings found that drought increased root:shoot ratio, and that seedling provenances exhibiting greater above‐ground growth succumbed to lethal drought more rapidly than smaller seedlings (Goodrich et al., 2016). The trends described by Goodrich et al. (2016) follow expectations from functional equilibrium theory, which asserts that proportionally greater plant biomass should accumulate in plant compartments (e.g., roots vs. shoots) that will most efficiently alleviate plant resource limitations (de la Mata et al., 2014; Van Noordwijk, 1998). Meanwhile, the present study found that warmer temperatures resulted in reduced total plant biomass, including reductions in both roots and shoots and an increased proportion of shoot biomass compared to root biomass. These findings, coupled with those from Goodrich et al. (2016), suggest that elevated temperature and drought exert opposite effects on *P*. *strobiformis* seedlings. But in natural forest systems, multiple resource limitations often co‐occur (Canham et al., 1996). Thus, the present study of the influence of warming on seed and seedling development provides indicators but not rules for how *P*. *strobiformis* can be expected to perform under future climatic conditions. Additionally, we acknowledge that climate change will result in warmer conditions not only for reproductive tissues, as our warming method achieved, but also warm all plant structures and tissues simultaneously, albeit not equally. However, we emphasize the importance of potential effects of climate change on reproductive processes of forest trees, as forest reproductive processes are thermally sensitive, and seed production strongly influences subsequent plant development and thus forest persistence (Cairney & Pullman, 2007; Gruwez et al., 2014).

The frequency and intensity of heat waves in the western USA has increased over recent decades (Mazdiyasni & Aghakouchak, 2015), and climate models project increases in mean annual air temperature of up to 4℃ in the southwestern USA by mid‐century (Elias et al., 2018). Recent declines in productivity and increased tree mortality in the southwestern USA coinciding with increasing regional drought severity may portend species extirpation and transition to novel ecosystem states (Williams et al., 2013). In the present study, warming during growth was associated with increased seedling *E*, leaf conductance, and midday stem water potential, but not increased root mass or root:shoot mass ratios. Climate warming may thus be expected to promote drought stress in wild *P*. *strobiformis* seedlings by reducing root growth and, concomitantly, the ability of seedlings to satisfy leaf‐level water demand through enhanced absorption of soil water (Ratzmann et al., 2019). Our results suggest that *P*. *strobiformis* in natural settings may respond to warming with decreased seedling emergence and increased seedling mortality, albeit with relatively diminished mortality if seed maturation occurs under elevated temperatures. Our findings also suggest that warming during early seedling growth may result in complex effects on water relations and oxidative stress resistance, where warming during early seedling growth may exacerbate the effects of drought but increase seedling oxidative stress resistance. The latter two findings illustrate potentially opposing consequences of climate warming that should be further explored in addition to seedling responses to embryo warming under temperatures that exceed the 2.1℃ that our in situ seed warming treatment achieved. While it is unclear how increased oxidative stress resistance might affect *P*. *strobiformis* in the wild, we speculate that natural populations of *P*. *strobiformis* may be destabilized if elevated temperatures lead to declines in seedling emergence and survival as shown in this ex situ study. Validation of results described here under natural settings is a crucial next step for developing expectations of the effects of climate warming on the persistence of *P*. *strobiformis* populations.

## CONFLICT OF INTEREST

The authors declare no conflict of interest.

## Supporting information

Fig S1

Table S1

Table S2

## Data Availability

The data that support the findings of this study are openly available in Open Science Framework under: Moler (2021, May 26). Moler et al. (2021) Plant‐Environment Interactions. Retrieved from osf.io/2p5vw.
