# Supplementary figures and images for: Plant developmental stage influences responses of Pinus strobiformis seedlings to experimental warming
Source: Plant Environ Interact. 2021 Jun 20;2(3):148–64. doi: 10.1002/pei3.10055 (PMC10168050; doi:10.1002/pei3.10055)

Scree plot

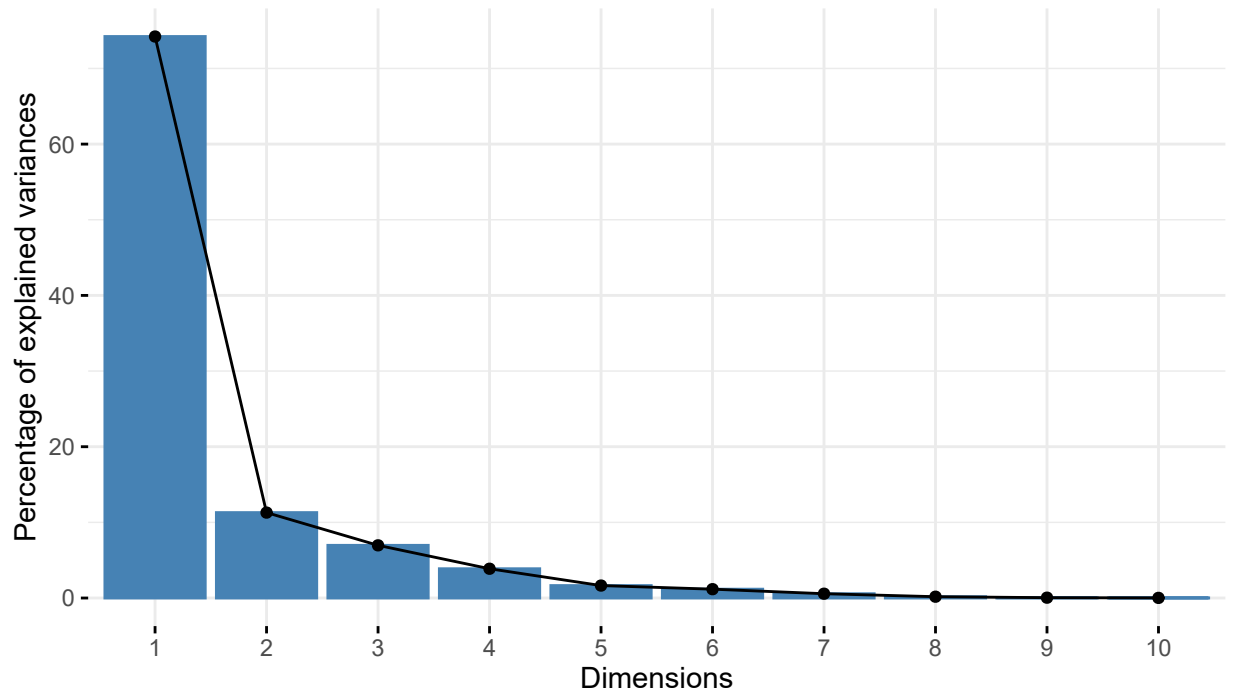

Supplement: Supplementary file 1 — Fig S1 [file PEI3-2-148-s002.pdf]
