# Supplementary material for: Plant developmental stage influences responses of Pinus strobiformis seedlings to experimental warming
Source: Plant Environ Interact. 2021 Jun 20;2(3):148–64. doi: 10.1002/pei3.10055 (PMC10168050; doi:10.1002/pei3.10055)
Supplement: Supplementary file 2 — Table S1 [file PEI3-2-148-s003.docx]

| **PC1** | | **PC2** | | **PC3** | |
| --- | --- | --- | --- | --- | --- |
| **Variable** | **Eigenvalue** | **Variable** | **Eigenvalue** | **Variable** | **Eigenvalue** |
| DD_18_03 | 0.57118 | Tmax08 | 3.53903 | PPT09 | 4.92380 |
| Tave03 | 0.57103 | Eref_sm | 3.35514 | PPT05 | 4.36952 |
| DD_18 | 0.57002 | EXT | 3.32474 | Rad06 | 3.82484 |
| DD_18_02 | 0.56935 | CMD | 3.29725 | CMD01 | 3.30117 |
| Tave02 | 0.56928 | MAP | 3.17924 | CMD_wt | 3.22664 |
| DD_0_01 | 0.56833 | Tmax07 | 3.12891 | CMD09 | 3.15293 |
| MAT | 0.56830 | Eref08 | 3.09449 | CMD02 | 3.12419 |
| DD_0_wt | 0.56823 | Tmax_sm | 2.81413 | Rad05 | 2.81766 |
| DD_0_02 | 0.56816 | Eref06 | 2.67700 | PAS08 | 2.78776 |
| DD_0 | 0.56779 | Eref07 | 2.64967 | PPT06 | 2.29772 |
| DD_18_06 | 0.56766 | CMD10 | 2.64665 | CMD11 | 1.98650 |
| Tave11 | 0.56726 | CMD_at | 2.46547 | CMD06 | 1.78284 |
| DD_18_11 | 0.56725 | PPT10 | 2.33249 | SHM | 1.66015 |
| DD_18_10 | 0.56713 | Eref09 | 2.31899 | DD18_03 | 1.57672 |
| Tave_wt | 0.56709 | PPT12 | 1.69696 | DD18_04 | 1.54330 |
| Tave10 | 0.56698 | CMD05 | 1.56877 | CMD03 | 1.51552 |
| DD_18_wt | 0.56690 | CMD08 | 1.55662 | DD18_07 | 1.29883 |
| DD_0_12 | 0.56590 | PPT_at | 1.41130 | PPT03 | 1.26827 |
| Tave06 | 0.56582 | PPT08 | 1.38597 | MSP | 1.25990 |
| DD5_06 | 0.56570 | Tmax09 | 1.21117 | Eref04 | 1.18208 |
| Tave_sp | 0.56522 | Eref_at | 1.17437 | PPT01 | 1.14587 |
| DD_0_03 | 0.56520 | SHM | 1.13823 | Eref_sp | 1.12525 |
| Tave12 | 0.56502 | PAS08 | 1.11578 | Eref05 | 1.10269 |
| DD_18_12 | 0.56498 | AHM | 1.07641 | PPT11 | 1.09228 |
| DD_18_sp | 0.56490 | CMD_sm | 1.02123 | Eref03 | 0.96651 |
| DD_18_at | 0.56450 | RH11 | 0.99710 | PPT_wt | 0.95658 |
| DD_0_11 | 0.56299 | Eref10 | 0.97927 | DD18_08 | 0.90827 |
| DD_18_01 | 0.56286 | CMD09 | 0.96974 | PPT_sm | 0.78012 |
| Tave_at | 0.56279 | CMD06 | 0.96731 | DD18_sm | 0.76602 |
| Tave01 | 0.56272 | RH12 | 0.90943 | NFFD01 | 0.73690 |
| MCMT | 0.56272 | RH10 | 0.86383 | NFFD12 | 0.73690 |
| Tave05 | 0.56262 | PAS_sm | 0.83167 | PPT02 | 0.73449 |
| DD_0_at | 0.56195 | RH | 0.81289 | Tave07 | 0.72726 |
| DD5_10 | 0.56195 | RH08 | 0.80017 | MWMT | 0.72726 |
| DD_0_sp | 0.56147 | RH05 | 0.79338 | DD18_09 | 0.71437 |
| DD_18_05 | 0.56078 | Tmax10 | 0.78607 | DD5_07 | 0.69894 |
| DD5_05 | 0.56077 | RH_sm | 0.78469 | NFFD_wt | 0.68944 |
| NFFD09 | 0.55985 | DD_18_07 | 0.76737 | DD18_sp | 0.68538 |
| Tmin03 | 0.55748 | DD_18_08 | 0.76386 | Rad08 | 0.68248 |
| NFFD_sm | 0.55559 | Tmax06 | 0.75853 | PAS09 | 0.64692 |
| DD5_sp | 0.55556 | RH06 | 0.74590 | Tmax04 | 0.64309 |
| DD5 | 0.55512 | RH_at | 0.74081 | SMRSPB | 0.62373 |
| NFFD06 | 0.55401 | DD5_08 | 0.73423 | RH04 | 0.62353 |
| NFFD05 | 0.55347 | Tave08 | 0.72908 | CMD_sp | 0.60589 |
| Tmin_sp | 0.55329 | CMD07 | 0.72719 | RH12 | 0.59986 |
| Tmin06 | 0.55318 | Tmax_at | 0.72708 | NFFD11 | 0.59691 |
| Tmin02 | 0.55262 | RH07 | 0.71949 | NFFD02 | 0.59651 |
| DD5_03 | 0.55085 | RH01 | 0.71868 | Rad_sm | 0.59036 |
| DD_18_04 | 0.55082 | RH04 | 0.70792 | Eref06 | 0.58332 |
| DD5_at | 0.55074 | RH_wt | 0.68593 | Eref10 | 0.57076 |
| NFFD_sp | 0.55060 | DD5_07 | 0.67582 | DD5_01 | 0.55554 |
| Tave04 | 0.54976 | RH09 | 0.67508 | RH11 | 0.55310 |
| NFFD10 | 0.54947 | RH_sp | 0.66645 | CMD_sm | 0.54016 |
| Tmin_sm | 0.54898 | Tave07 | 0.64677 | CMD04 | 0.53106 |
| Tmin05 | 0.54852 | MWMT | 0.64677 | Eref_at | 0.52155 |
| bFFP | 0.54801 | CMD02 | 0.62870 | RH05 | 0.52142 |
| Tmin_wt | 0.54704 | CMD_wt | 0.61959 | DD18_05 | 0.50897 |
| Tmin04 | 0.54690 | DD18_08 | 0.59597 | PPT07 | 0.50633 |
| FFP | 0.54667 | PPT_sm | 0.58893 | RH_sp | 0.49694 |
| NFFD | 0.54478 | CMD01 | 0.58664 | Tave08 | 0.49223 |
| NFFD_at | 0.54190 | RH02 | 0.58564 | Rad_sp | 0.48687 |
| DD_0_04 | 0.54162 | MSP | 0.56389 | DD5_08 | 0.47838 |
| Tmin12 | 0.54154 | CMD11 | 0.53227 | DD18_10 | 0.46988 |
| Tmin01 | 0.54102 | Tmax03 | 0.53055 | Tmax07 | 0.45108 |
| Tmin10 | 0.53940 | DD18_07 | 0.51314 | Eref | 0.44073 |
| PAS03 | 0.53904 | TD | 0.45078 | DD_18_07 | 0.43075 |
| Tmin09 | 0.53779 | PPT05 | 0.44658 | RH01 | 0.42232 |
| PAS01 | 0.53731 | Eref | 0.43075 | DD5_12 | 0.41715 |
| EMT | 0.53685 | RH03 | 0.42593 | Eref11 | 0.41671 |
| NFFD04 | 0.53682 | PPT07 | 0.40615 | Tmax_sp | 0.41504 |
| DD_18_09 | 0.53682 | Eref03 | 0.37301 | RH_wt | 0.41133 |
| eFFP | 0.53645 | Eref05 | 0.36366 | Tmin07 | 0.40245 |
| Tmin_at | 0.53599 | DD18_11 | 0.35383 | RH02 | 0.39174 |
| PAS_sp | 0.53481 | PPT11 | 0.34572 | Rad10 | 0.38898 |
| Tave09 | 0.53419 | Tmax01 | 0.33457 | CMD07 | 0.38484 |
| DD5_04 | 0.53416 | NFFD08 | 0.30866 | DD_18_08 | 0.38468 |
| DD5_09 | 0.53356 | Eref_sp | 0.30665 | PPT12 | 0.36361 |
| DD_0_10 | 0.53255 | Tmax11 | 0.30472 | RH03 | 0.35989 |
| PAS | 0.53241 | Rad10 | 0.30156 | DD5_wt | 0.35666 |
| Tmin07 | 0.53213 | Eref11 | 0.28035 | DD18 | 0.35500 |
| PAS02 | 0.53112 | PAS09 | 0.26617 | Tmin08 | 0.35006 |
| Tmin11 | 0.53054 | Tmax_wt | 0.25837 | Eref02 | 0.34937 |
| PAS_wt | 0.52913 | PPT06 | 0.24071 | RH_at | 0.32870 |
| DD5_11 | 0.52764 | DD_18_sm | 0.23743 | PAS_sm | 0.31783 |
| PAS_at | 0.52589 | DD5_sm | 0.23528 | PPT10 | 0.30817 |
| Tmin08 | 0.52567 | Tmax12 | 0.23477 | DD18_at | 0.30318 |
| PAS11 | 0.52556 | NFFD11 | 0.23240 | RH | 0.30236 |
| DD18_06 | 0.52522 | NFFD02 | 0.23065 | Tmax05 | 0.30009 |
| DD5_02 | 0.52231 | Tave_sm | 0.22278 | Tmax03 | 0.29722 |
| Tmax02 | 0.51949 | DD18_sp | 0.22062 | Eref09 | 0.28720 |
| DD18_at | 0.51607 | Rad06 | 0.22015 | DD5_11 | 0.27514 |
| NFFD03 | 0.51592 | DD18_04 | 0.21822 | DD5_02 | 0.27352 |
| PPT04 | 0.51525 | PAS12 | 0.21626 | NFFD03 | 0.26754 |
| PAS04 | 0.51340 | NFFD01 | 0.20094 | CMD05 | 0.26306 |
| DD_18_sm | 0.51122 | NFFD12 | 0.20094 | RH09 | 0.25700 |
| DD18 | 0.50692 | Eref04 | 0.19389 | DD18_06 | 0.24981 |
| DD5_wt | 0.50660 | DD18_10 | 0.18568 | Tave_sm | 0.24973 |
| Tmax_wt | 0.50598 | Tmax02 | 0.18553 | Rad02 | 0.24549 |
| Eref_wt | 0.50529 | Tmin11 | 0.17529 | DD5_09 | 0.24379 |
| CMD04 | 0.50444 | NFFD04 | 0.17494 | DD5_sm | 0.24251 |
| Tmax11 | 0.50440 | NFFD_wt | 0.17062 | Tave09 | 0.23960 |
| Tave_sm | 0.50436 | DD18_sm | 0.16825 | Tmin09 | 0.23665 |
| DD5_sm | 0.50399 | DD18_05 | 0.16440 | PPT08 | 0.22823 |
| Eref02 | 0.50364 | CMD_sp | 0.16119 | PAS01 | 0.21480 |
| Tmax12 | 0.50283 | Tmax_sp | 0.15573 | Tmax08 | 0.21459 |
| Tmax05 | 0.49913 | Tmin10 | 0.14302 | DD_18_09 | 0.19895 |
| Tmax_sp | 0.49670 | bFFP | 0.14297 | Rad04 | 0.19244 |
| PPT_sp | 0.49531 | PAS10 | 0.14175 | RH10 | 0.18925 |
| Tmax01 | 0.48746 | Eref02 | 0.13997 | Tmin_sm | 0.18894 |
| PAS12 | 0.48590 | Rad09 | 0.13747 | TD | 0.18854 |
| DD18_09 | 0.48585 | NFFD10 | 0.12958 | Rad_at | 0.18524 |
| DD5_12 | 0.48514 | NFFD_at | 0.12866 | EMT | 0.18118 |
| SMRSPB | 0.48104 | Tmin04 | 0.12852 | eFFP | 0.17511 |
| Tmax04 | 0.48058 | NFFD_sp | 0.12622 | Tmin_at | 0.16418 |
| CMD_sp | 0.48042 | FFP | 0.12578 | RH06 | 0.15677 |
| DD5_01 | 0.47433 | NFFD | 0.12475 | Tmin11 | 0.15651 |
| PPT02 | 0.47267 | Tmin12 | 0.12463 | DD5_at | 0.15536 |
| NFFD11 | 0.46947 | Tmin_at | 0.12426 | PPT_sp | 0.15113 |
| NFFD_wt | 0.46920 | Tmin05 | 0.12342 | Eref_wt | 0.14759 |
| NFFD02 | 0.46814 | Tmin01 | 0.12241 | PAS02 | 0.14418 |
| RH03 | 0.46653 | Rad_at | 0.11878 | CMD08 | 0.14281 |
| Eref11 | 0.46566 | DD_0_05 | 0.11672 | DD_18_sm | 0.14076 |
| PAS10 | 0.46324 | EMT | 0.11528 | PAS03 | 0.14055 |
| Tmax_at | 0.45836 | Tmax05 | 0.11515 | RH_sm | 0.13672 |
| NFFD01 | 0.45651 | SMRSPB | 0.11447 | PAS_sp | 0.12929 |
| NFFD12 | 0.45651 | NFFD03 | 0.11446 | Tmin01 | 0.12686 |
| DD18_sm | 0.45592 | Tmin_wt | 0.10741 | Tmax09 | 0.12441 |
| DD18_10 | 0.45451 | Tmin_sp | 0.10114 | PAS11 | 0.12329 |
| PPT07 | 0.45077 | PAS_at | 0.09307 | MAR | 0.12259 |
| Tmax10 | 0.45065 | NFFD05 | 0.09177 | PAS05 | 0.11930 |
| RH07 | 0.45060 | eFFP | 0.09116 | Tmax_sm | 0.11758 |
| PPT01 | 0.44974 | Tmin02 | 0.08960 | Tmin12 | 0.11521 |
| Eref01 | 0.44907 | PPT_wt | 0.08828 | PPT04 | 0.11486 |
| Tmax03 | 0.44640 | PPT_sp | 0.08650 | Tmax02 | 0.11457 |
| PAS05 | 0.44614 | Tmin06 | 0.08345 | PAS04 | 0.11435 |
| Eref12 | 0.44599 | PAS04 | 0.07705 | DD_0_04 | 0.10927 |
| PPT_wt | 0.44413 | Tmax04 | 0.07189 | PAS_wt | 0.10833 |
| RH02 | 0.44088 | PAS05 | 0.07003 | Rad11 | 0.10569 |
| RH_sm | 0.44064 | Tmin03 | 0.06459 | PAS | 0.10486 |
| Eref | 0.44035 | Tmin09 | 0.05707 | Tave04 | 0.10157 |
| RH06 | 0.43479 | Rad08 | 0.05593 | DD_18_04 | 0.09872 |
| TD | 0.43456 | Rad11 | 0.05297 | NFFD_at | 0.09392 |
| RH09 | 0.43439 | PAS11 | 0.04842 | Tmin_wt | 0.09349 |
| PPT03 | 0.43330 | CMD04 | 0.04543 | RH07 | 0.09188 |
| Tmax06 | 0.43131 | DD18 | 0.04511 | PPT_at | 0.08878 |
| DD18_05 | 0.42880 | PAS | 0.04334 | Tmin10 | 0.08842 |
| RH08 | 0.42765 | PPT02 | 0.04186 | DD_0_05 | 0.08390 |
| RH_at | 0.42705 | MAR | 0.04125 | DD5_04 | 0.08066 |
| RH_wt | 0.42657 | PPT01 | 0.04083 | NFFD | 0.08018 |
| RH_sp | 0.42383 | Rad03 | 0.03960 | Tmax11 | 0.07876 |
| DD_0_05 | 0.42056 | PAS_sp | 0.03843 | Tmax_wt | 0.07719 |
| RH01 | 0.42046 | PAS_wt | 0.03834 | Eref_sm | 0.07709 |
| RH | 0.42025 | Rad_sm | 0.03715 | Rad09 | 0.07697 |
| CMD07 | 0.41822 | DD5_04 | 0.03680 | PAS10 | 0.07656 |
| Eref04 | 0.41659 | PAS03 | 0.03392 | Tmax12 | 0.07300 |
| RH10 | 0.41472 | Eref12 | 0.03206 | DD5 | 0.07153 |
| Eref_sp | 0.40866 | DD5_11 | 0.03061 | Tave_at | 0.07009 |
| PPT_sm | 0.40559 | DD_18_09 | 0.03003 | Tmin02 | 0.06410 |
| DD_18_08 | 0.40134 | Tave09 | 0.02972 | RH08 | 0.06314 |
| Eref03 | 0.40114 | PAS02 | 0.02841 | Rad12 | 0.06102 |
| DD5_08 | 0.40044 | DD5_02 | 0.02837 | Tmin06 | 0.06096 |
| Tave08 | 0.39862 | Eref01 | 0.02804 | DD_18_at | 0.06065 |
| RH04 | 0.39708 | DD5_09 | 0.02582 | NFFD08 | 0.05907 |
| CMD03 | 0.39338 | Rad01 | 0.02429 | CMD10 | 0.05791 |
| Rad02 | 0.39219 | PPT03 | 0.02322 | DD5_10 | 0.05714 |
| AHM | 0.39148 | Tmin_sm | 0.02240 | Eref12 | 0.05680 |
| RH05 | 0.38982 | DD_0_04 | 0.02207 | PAS_at | 0.05638 |
| Rad_wt | 0.38806 | CMD03 | 0.02097 | DD5_03 | 0.04983 |
| DD_18_07 | 0.38736 | DD_0_10 | 0.01988 | Tmin03 | 0.04808 |
| Rad04 | 0.38680 | Rad_wt | 0.01832 | AHM | 0.04451 |
| PPT11 | 0.38569 | Rad05 | 0.01646 | Eref01 | 0.04114 |
| DD18_sp | 0.38305 | DD5_sp | 0.01609 | Tmax01 | 0.04007 |
| Tave07 | 0.38267 | DD5 | 0.01523 | Eref08 | 0.03777 |
| MWMT | 0.38267 | DD5_01 | 0.01362 | FFP | 0.03757 |
| DD5_07 | 0.38247 | DD_0_sp | 0.01284 | Tmin05 | 0.03585 |
| Rad11 | 0.37992 | DD_0_03 | 0.01239 | Rad07 | 0.03429 |
| Rad01 | 0.37634 | Rad02 | 0.01221 | DD_0_sp | 0.03320 |
| DD18_08 | 0.37376 | Rad12 | 0.01169 | DD_18_10 | 0.03260 |
| Rad12 | 0.36848 | PPT09 | 0.01107 | DD_18_11 | 0.03034 |
| RH12 | 0.36709 | Tave04 | 0.01084 | NFFD05 | 0.03014 |
| MSP | 0.36578 | DD5_12 | 0.01034 | EXT | 0.02956 |
| Eref05 | 0.36396 | DD_18_04 | 0.00993 | Tave10 | 0.02807 |
| CMD_sm | 0.36230 | DD_0_at | 0.00905 | DD_18_01 | 0.02805 |
| Eref10 | 0.36054 | DD5_05 | 0.00853 | Tave11 | 0.02800 |
| Rad03 | 0.36010 | Rad_sp | 0.00839 | CMD | 0.02723 |
| RH11 | 0.35791 | DD5_wt | 0.00830 | Tave06 | 0.02506 |
| DD18_07 | 0.35284 | DD18_at | 0.00787 | DD5_06 | 0.02442 |
| PAS09 | 0.34945 | Tave05 | 0.00736 | MCMT | 0.02438 |
| Rad08 | 0.34650 | Tave11 | 0.00605 | Tave01 | 0.02438 |
| NFFD08 | 0.34307 | DD_18_11 | 0.00566 | MAP | 0.02353 |
| Eref_at | 0.34186 | MAT | 0.00507 | PAS12 | 0.02319 |
| PPT08 | 0.34041 | NFFD_sm | 0.00429 | Tave_sp | 0.02227 |
| MAR | 0.32830 | DD18_06 | 0.00421 | MAT | 0.02192 |
| Rad_sp | 0.32459 | PPT04 | 0.00385 | DD_0_03 | 0.02143 |
| PPT06 | 0.31715 | DD5_03 | 0.00378 | DD_18_sp | 0.02103 |
| Tmax09 | 0.31258 | NFFD06 | 0.00339 | Tmin_sp | 0.01929 |
| Rad_at | 0.30793 | DD5_06 | 0.00298 | DD5_sp | 0.01608 |
| CMD08 | 0.30376 | DD_0_11 | 0.00292 | NFFD10 | 0.01579 |
| DD18_04 | 0.28676 | DD_18 | 0.00288 | NFFD09 | 0.01463 |
| CMD05 | 0.28373 | Tave06 | 0.00278 | DD_0_at | 0.01334 |
| PPT_at | 0.26787 | Tave_sp | 0.00278 | NFFD_sm | 0.01303 |
| DD18_03 | 0.25252 | DD_18_06 | 0.00272 | Tave05 | 0.01224 |
| CMD06 | 0.25117 | Tave02 | 0.00248 | DD_18_wt | 0.01215 |
| Rad10 | 0.24135 | DD_0 | 0.00188 | DD_18 | 0.01123 |
| SHM | 0.23746 | Tave10 | 0.00175 | CMD_at | 0.01072 |
| Rad09 | 0.23199 | NFFD09 | 0.00146 | DD_0_02 | 0.01039 |
| DD18_11 | 0.17763 | DD_18_02 | 0.00144 | DD_18_12 | 0.01030 |
| PPT12 | 0.16107 | DD_18_10 | 0.00143 | NFFD_sp | 0.01017 |
| CMD11 | 0.14511 | DD_18_sp | 0.00137 | Tave_wt | 0.00971 |
| CMD10 | 0.14268 | Tave03 | 0.00111 | Tave12 | 0.00900 |
| Eref07 | 0.14119 | Eref_wt | 0.00104 | Tmax06 | 0.00863 |
| CMD_at | 0.13840 | DD5_at | 0.00103 | Tmax10 | 0.00847 |
| Rad_sm | 0.12318 | DD_18_wt | 0.00097 | DD_0 | 0.00830 |
| Rad07 | 0.12211 | DD_18_12 | 0.00094 | DD5_05 | 0.00824 |
| Rad05 | 0.10662 | Tave_at | 0.00093 | DD_18_05 | 0.00824 |
| Tmax_sm | 0.10123 | Tmin08 | 0.00091 | Rad01 | 0.00816 |
| Eref09 | 0.08551 | Tave_wt | 0.00090 | DD_0_12 | 0.00794 |
| Eref08 | 0.08384 | Tmin07 | 0.00090 | DD_0_11 | 0.00662 |
| CMD01 | 0.07265 | DD_18_05 | 0.00088 | DD_18_06 | 0.00570 |
| Eref06 | 0.07160 | DD_0_02 | 0.00087 | DD_0_wt | 0.00431 |
| Rad06 | 0.07123 | DD_18_03 | 0.00084 | NFFD04 | 0.00388 |
| PPT10 | 0.06962 | DD_18_at | 0.00080 | Tave02 | 0.00312 |
| CMD_wt | 0.06187 | DD_18_01 | 0.00064 | Tmax_at | 0.00271 |
| CMD02 | 0.05469 | Tave12 | 0.00064 | bFFP | 0.00268 |
| PPT09 | 0.05448 | MCMT | 0.00034 | Rad_wt | 0.00252 |
| CMD | 0.04938 | Tave01 | 0.00034 | DD_18_02 | 0.00219 |
| Eref_sm | 0.03863 | DD_0_wt | 0.00033 | DD18_11 | 0.00171 |
| MAP | 0.03501 | DD_0_01 | 0.00031 | DD_0_10 | 0.00159 |
| PPT05 | 0.02957 | Rad04 | 0.00026 | NFFD06 | 0.00098 |
| EXT | 0.02372 | Rad07 | 0.00020 | Rad03 | 0.00096 |
| PAS08 | 0.00507 | DD18_09 | 0.00019 | DD_0_01 | 0.00051 |
| Tmax08 | 0.00482 | DD_0_12 | 0.00017 | Tmin04 | 0.00037 |
| CMD09 | 0.00067 | PAS01 | 0.00016 | Tave03 | 0.00024 |
| Tmax07 | 0.00011 | DD5_10 | 0.00007 | Eref07 | 0.00008 |
| PAS_sm | 0.00003 | DD18_03 | 0.00001 | DD_18_03 | 0.00002 |
