# Supplementary material for: Plant developmental stage influences responses of Pinus strobiformis seedlings to experimental warming
Source: Plant Environ Interact. 2021 Jun 20;2(3):148–64. doi: 10.1002/pei3.10055 (PMC10168050; doi:10.1002/pei3.10055)
Supplement: Supplementary file 3 — Table S2 [file PEI3-2-148-s001.docx]

Supplementary Table 2. AIC comparisons for models with significant fixed effects. Best-performing models are shown with ∆AIC value = 0. Model parsimony reduces with ∆AIC value size. Interactive models include interaction terms between each predictor, which tests predictor interactions in a step-wise fashion including all possible combinations. If significant interactions were not detected, AIC values of interactive models were not compared.

| **Full Model Example: Response ~ Chamber Treatment + Embryo Warming Treatment + Seedmass + Climate + (random = Site\Family)** | |
| --- | --- |
| **AIC Model Selection for Ten-Day ψ** | **∆AIC** |
| Additive without climate | 0 |
| Full additive | 3.6 |
| Additive without climate or seedmass | 9.5 |
| Additive without seedmass | 12.8 |
| **AIC Model Selection for Mortality** | **∆AIC** |
| Additive without climate | 0 |
| Full additive | 1.8 |
| Additive without climate or seedmass | 3.8 |
| Additive without seedmass | 5.8 |
| **AIC Model Selection for Days to Mortality** | **∆AIC** |
| Full interactive | 0 |
| Interactive without climate | 145.5 |
| Interactive without seedmass | 167.2 |
| Additive without climate | 252.9 |
| Full additive | 253 |
| Interactive without climate or seedmass | 263.2 |
| Additive without climate or seedmass | 318.7 |
| Additive without seedmass | 320.3 |
| **AIC Model Selection for Proportion Emerged** | **∆AIC** |
| Full interactive | 0 |
| Full additive | 1.6 |
| Additive without climate | 3.1 |
| Interactive without climate | 4.8 |
| Interactive without seedmass | 1314.3 |
| Additive without climate or seedmass | 1329.8 |
| Additive without seedmass | 1331.8 |
| Interactive without climate or seedmass | 1332.2 |
| **AIC Model Selection for DRC** | **∆AIC** |
| Additive without climate | 0 |
| Full additive | 13.8 |
| Additive without climate or seedmass | 97.5 |
| Additive without seedmass | 108 |
| **AIC Model Selection for Total Seedling Mass** | **∆AIC** |
| Additive without climate | 0 |
| Full additive | 12.1 |
| Additive without climate or seedmass | 56.2 |
| Additive without seedmass | 67.1 |
| **AIC Model Selection for Cotyledon Number** | **∆AIC** |
| Additive without climate | 0 |
| Full additive | 8.2 |
| Additive without climate or seedmass | 77.9 |
| Additive without seedmass | 84.3 |
| **AIC Model Selection for Cotyledon Length** | **∆AIC** |
| Additive without climate | 0 |
| Full additive | 12.5 |
| Additive without climate or seedmass | 52 |
| Additive without seedmass | 62.4 |
| **AIC Model Selection for Root : Shoot Mass Ratio** | **∆AIC** |
| Additive without climate or seedmass | 0 |
| Additive without climate | 2.8 |
| Additive without seedmass | 9.2 |
| Full additive | 11.7 |
| **AIC Model Selection for Slenderness** | **∆AIC** |
| Additive without climate or seedmass | 0 |
| Additive without climate | 1.2 |
| Additive without seedmass | 6.3 |
| Full additive | 7.3 |
| **AIC Model Selection for Root Mass** | **∆AIC** |
| Additive without climate | 0 |
| Full additive | 11.5 |
| Additive without climate or seedmass | 39.2 |
| Additive without seedmass | 50.4 |
| **AIC Model Selection for Shoot Mass** | **∆AIC** |
| Additive without climate | 0 |
| Full additive | 15.9 |
| Additive without climate or seedmass | 63.7 |
| Additive without seedmass | 75.5 |
| **AIC Model Selection for YII 24 hr after Paraquat Treatment** | **∆AIC** |
| Additive without climate or seedmass | 0 |
| Additive without climate | 4.3 |
| Additive without seedmass | 14.8 |
| Full additive | 19 |
| Interactive without climate | 34.6 |
| Interactive without climate or seedmass | 35.5 |
| Interactive without seedmass | 179.9 |
| Full interactive | 225.7 |
| **AIC Model Selection for YII 48 hr after Paraquat Treatment** | **∆AIC** |
| Additive without climate | 0 |
| Additive without climate or seedmass | 0.1 |
| Interactive without climate | 3.7 |
| Interactive without climate or seedmass | 10.6 |
| Additive without seedmass | 63.5 |
| Full additive | 70.3 |
| Interactive without seedmass | 150.8 |
| Full interactive | 187.8 |
| **AIC Model Selection for Leaf Conductance** | **∆AIC** |
| Additive without climate or seedmass | 0 |
| Additive without climate | 5.6 |
| Additive without seedmass | 16.1 |
| Full additive | 22.2 |
| **AIC Model Selection for *E*** | **∆AIC** |
| Additive without climate | 0 |
| Additive without climate or seedmass | 0.8 |
| Full additive | 10.2 |
| Additive without seedmass | 11 |
| **AIC Model Selection for RWC** | **∆AIC** |
| Additive without climate or seedmass | 0 |
| Additive without climate | 2.1 |
| Additive without seedmass | 9 |
| Full additive | 11.3 |
